# Toward Understanding Phage:Host Interactions in the Rumen; Complete Genome Sequences of Lytic Phages Infecting Rumen Bacteria

**DOI:** 10.3389/fmicb.2017.02340

**Published:** 2017-12-05

**Authors:** Rosalind A. Gilbert, William J. Kelly, Eric Altermann, Sinead C. Leahy, Catherine Minchin, Diane Ouwerkerk, Athol V. Klieve

**Affiliations:** ^1^Department of Agriculture and Fisheries, EcoSciences Precinct, Brisbane, QLD, Australia; ^2^Queensland Alliance for Agriculture and Food Innovation, University of Queensland, St Lucia, QLD, Australia; ^3^Donvis Ltd., Palmerston North, New Zealand; ^4^AgResearch Limited, Grasslands Research Centre, Palmerston North, New Zealand; ^5^Riddet Institute, Massey University, Palmerston North, New Zealand; ^6^New Zealand Agricultural Greenhouse Gas Research Centre, Palmerston North, New Zealand; ^7^School of Agriculture and Food Sciences, University of Queensland, Gatton Campus, Gatton, QLD, Australia

**Keywords:** bacteriophage, phage, rumen, *Streptococcus*, *Bacteroides*, *Ruminococcus*, Siphoviridae, Podoviridae

## Abstract

The rumen is known to harbor dense populations of bacteriophages (phages) predicted to be capable of infecting a diverse range of rumen bacteria. While bacterial genome sequencing projects are revealing the presence of phages which can integrate their DNA into the genome of their host to form stable, lysogenic associations, little is known of the genetics of phages which utilize lytic replication. These phages infect and replicate within the host, culminating in host lysis, and the release of progeny phage particles. While lytic phages for rumen bacteria have been previously isolated, their genomes have remained largely uncharacterized. Here we report the first complete genome sequences of lytic phage isolates specifically infecting three genera of rumen bacteria: *Bacteroides, Ruminococcus*, and *Streptococcus*. All phages were classified within the viral order Caudovirales and include two phage morphotypes, representative of the Siphoviridae and Podoviridae families. The phage genomes displayed modular organization and conserved viral genes were identified which enabled further classification and determination of closest phage relatives. Co-examination of bacterial host genomes led to the identification of several genes responsible for modulating phage:host interactions, including CRISPR/*Cas* elements and restriction-modification phage defense systems. These findings provide new genetic information and insights into how lytic phages may interact with bacteria of the rumen microbiome.

## Introduction

The rumen contains dense and highly diverse populations of bacteriophages (phages). Rumen phage populations have been examined using culture-independent techniques such as morphological surveys (electron microscopy), genome length profiling (pulsed field gel electrophoresis) and more recently, viral metagenomics (sequencing of viral community DNA) (Paynter et al., 1969; Klieve and Bauchop, 1988; Klieve and Swain, 1993; Berg Miller et al., 2012; Ross et al., 2013). Culture-based techniques have also been employed for studying rumen phage populations, where successful phage infection and replication is indicated by the formation of clearing zones or plaques in otherwise confluent layers of bacterial growth (Klieve, 2005). This approach tends to detect phages able to undergo the lytic cycle of phage infection where phage particles attach to and replicate in susceptible bacteria without forming a stable genetic association with their host (lysogeny) (Ackermann and DuBow, 1987). Culture-based techniques cannot fully encompass the viral diversity of the rumen source material, instead they capture viable phages which can successfully infect and replicate in the bacterial strains employed in the study.

The first isolation of lytic phages from rumen fluid was reported in 1966 using rumen fluid from cattle as source material and rumen-derived isolates of *Serratia* and *Streptococcus* as bacterial hosts (Adams et al., 1966). Following this initial report, rumen fluid was used as a source of lytic phages able to infect a range of predominant rumen bacterial genera for example *Streptococcus, Bacteroides*, and *Ruminococcus* (Iverson and Millis, 1976a; Tarakanov, 1976; Klieve et al., 1991, 2004; Styriak et al., 1994). While many rumen phages were isolated and stored in culture collections, particularly in the 1970's and 1980's (Gilbert and Klieve, 2015), only those phages which could be potentially employed in applications such as genetic engineering and phage therapy were characterized beyond an initial assessment of morphology.

This study describes the genome sequences of five lytic phages capable of infecting rumen bacteria. These phages have all been previously described and classified as Caudovirales, having double-stranded DNA genomes and characteristic head-tail morphology. Three of the phages, ϕBrb01 and ϕBrb02 infecting strains of *Bacteroides* sp. (Klieve et al., 1991) and ϕSb01 (Klieve and Bauchop, 1991) infecting strains of *Streptococcus* classified within the *Streptococcus bovis*/*Streptococcus equinus* complex (SBSEC) (Jans et al., 2015), form particles with long, flexible tails characteristic of the family Siphoviridae. The further two phages, ϕRa02 and ϕRa04 infecting *Ruminococcus albus* AR67 (Klieve et al., 2004) form smaller particles with short tails and have been classified within the Podoviridae family.

The phages ϕBrb01 and ϕBrb02 have been the most genetically characterized, with restriction mapping experiments showing both genomes to be circularly permuted and terminally redundant (Klieve et al., 1991) resulting in the formation of phage particles containing variable lengths of genomic DNA. Rumen phage:host interactions have also been reported with investigations into the host range of the *Bacteroides* phages ϕBrb01 and ϕBrb02 and the *Streptococcus* phage ϕSb01, indicating that each of these Siphoviridae could utilize multiple strains within a single bacterial species as hosts (Klieve et al., 1991, 1999). Physical mechanisms for the development of phage resistance have also been observed, with the formation of a thick, polysaccharide capsule conferring *Bacteroides* resistance to infection by ϕBrb01 (Klieve et al., 1991).

This study addresses the paucity of information available for rumen phages in current sequence databases and represents the first report of complete genome sequences for lytic phages infecting rumen bacteria. It also provides the first genome sequences of lytic phages infecting the genus *Ruminococcus*. The genome sequences of the host bacteria were also analyzed in order to determine how these phages interact with their host and provide new insights into the defense mechanisms rumen bacteria may employ to counteract phage infection.

## Materials and methods

### Phage cultivation and phage DNA extraction

Phages and respective host bacteria are detailed in Table 1. All cultivation of bacteria and phage isolates was undertaken in a Physical Containment Level 2, Australian Government Office of the Gene Technology Regulator (OTGR) certified laboratory facility using biosecurity and institutional safety procedures required for these organisms and for products derived from these organisms, including DNA (Biohazard Risk Group 1 and 2—Animal origin). Although three of the phages have been previously shown to infect multiple host strains, *S. equinus* 2B (also classified as *S. bovis* 2B) was used as the host strain for cultivation of ϕSb01 and *Bacteroides* sp. AR20 was used as the host strain for ϕBrb01 and ϕBrb02 and *R. albus* AR67 was used as the host strain for ϕRa02 and ϕRa04. Rumen bacterial host strains were cultivated using anaerobic techniques (Hungate et al., 1964) and a rumen fluid-based growth medium (Klieve et al., 1989). Phages were propagated according to previously described methods (Klieve, 2005) with phage infection times adjusted to account for differential growth rates (2 h for *S. equinus* 2B and 4–6 h for *R. albus* AR67 and *Bacteroides* sp. AR20). Phage particles were harvested 24 h post-infection by differential centrifugation, filtration through 0.45 and 0.22 μm low protein binding filter units (Millipore) (Klieve, 2005) and precipitated with 20% (w/v) PEG6000 and 25 M NaCl at 4°C for a minimum of 1 h. Phage particles were pelleted by ultracentrifugation at 52,350 × g for 2 h at 4°C (50.2Ti rotor, Optima^TM^ L-100 XP Ultracentrifuge, Beckmann Coulter) and phage DNA extracted (Klieve and Gilbert, 2005).

**Table 1:** Summary of original phage descriptions (classification according to viral particle morphology, genome lengths calculated by restriction enzyme digestion of phage genomic DNA and particle dimensions) and description of bacterial host strains.

**Phage**	**Classification**	**Genome length (kb)**	**Phage particle dimensions**	**Phage isolation source (Reference)**	**Bacterial host/s**	**Bacterial host/s isolation source (Reference)**
ϕBrb01	Siphoviridae	33.91 ± 2.41	Head 55–60 nm, tail 105 × 9 nm	Municipal sewage (Klieve et al., 1991)	*Bacteroides* sp. AR20^***^, *Bacteroides* (*Prevotella*) *ruminicola* ss *brevis* AR22, AR23	Ovine rumen fluid (Klieve et al., 1989)
ϕBrb02	Siphoviridae	33.02 ± 1.16	Head 55–60 nm, tail 105 × 9 nm	Municipal sewage (Klieve et al., 1991)	*Bacteroides* sp. AR20^***^, *Bacteroides* (*Prevotella*) *ruminicola* ss *brevis* AR7, AR22, AR23	Ovine rumen fluid (Klieve et al., 1989)
ϕRa02	Podoviridae	12.8	Head 25 nm, tail 18 nm^*^	Bovine fecal waste material (Klieve et al., 2004)	*Ruminococcus albus* AR67	Ovine rumen fluid (Klieve et al., 1989)
ϕRa04	Podoviridae	14.0	Head 25 nm, tail 18 nm^*^	Bovine faecal waste material (Klieve et al., 2004)	*Ruminococcus albus* AR67	Ovine rumen fluid (Klieve et al., 1989)
ϕSb01	Siphoviridae	30.9 kb ± 4.4	Head 60 nm, tail 110 × 7 nm	Bovine rumen fluid (Klieve and Bauchop, 1991)	*Streptococcus bovis/equinus* 2B, *S. equinus* Sb04, Sb17	NS^**^ (Iverson and Millis, 1976b), ovine rumen fluid; bovine rumen fluid (Klieve et al., 1999)

* Tail dimension estimated from TEM image presented in the Corrigendum to original phage isolation publication (Klieve et al., 2006);

** *Not stated (NS) in original isolation reference (Iverson and Millis, Personal Communication)*;

*** *Bacteroides sp. strain AR20 was previously classified as Bacteroides ruminicola ss brevis, Prevotella ruminicola ss brevis in the Cytophaga-Flavobacterium-Bacteroides (CFB) group bacteria and Bacteroides thetaiotaomicro*.

### Sequencing and assembly

Genome sequences were generated at the U.S. Department of Energy, Joint Genome Institute (JGI). An Illumina standard shotgun library was constructed and sequenced using the Illumina HiSeq 2000 platform (2 × 151 bp read lengths with at least 50,000 reads generated per phage). All raw Illumina sequence data was quality filtered with DUK, a sequence filtering program developed at JGI, which removes Illumina sequencing and library preparation artifacts (Mingkun, L., Copeland, A. and Han, J., unpublished). Following the JGI standard protocol, genome assembly involved the following steps (1) quality filtered reads were assembled using Velvet (version 1.2.07) (Zerbino and Birney, 2008); (2) 1–3 kb simulated paired end reads were created from Velvet contigs using wgsim (https://github.com/lh3/wgsim); (3) reads were assembled with simulated read pairs using Allpaths–LG (version r46652) (Gnerre et al., 2011). For all phages sequenced, a minimum assembly input read coverage of 380 x was obtained (based on the number of quality-filtered reads) and each respective phage assembly resulted in one contig in one scaffold.

### Annotation

Open reading frames were determined using Glimmer version 3 (Delcher et al., 2007) within GAMOLA2 (Altermann et al., 2017) and Prodigal version 2.6.2, (Hyatt et al., 2010) within Prokka version 1.1 (Seemann, 2014). Sequence homologies were identified by GAMOLA2 using the protein model databases Pfam-A (release 30.0), TIGRfam 15.0, and COG2014 (Haft et al., 2013; Galperin et al., 2015; Finn et al., 2016), and Prokka version 1.1 using the protein model databases Pfam, COG clusters, HAMAP, Resfams, TIGRfam 15.0, and dbCAN v4 (Yin et al., 2012; Haft et al., 2013; Galperin et al., 2015; Gibson et al., 2015; Pedruzzi et al., 2015; Finn et al., 2016). The putative function of predicted ORF's were also assigned based on homologies to proteins identified in BLASTP and BLASTX searches (BLAST+ version 2.2.31, Camacho et al., 2009) using the National Center for Bioinformatic Information (NCBI) virus reference sequence database (update 11th January, 2017) containing 8321 virus genome sequences and the NCBI-nr database (January 2017 update), with an e-value threshold of 10^−3^. Annotations generated by Prokka and GAMOLA2 and additional BLAST searches were merged using Geneious R9 (Kearse et al., 2012). Transmembrane proteins, signal peptides and tRNA elements were identified by GAMOLA2 and Prokka. Predicted transmembrane proteins were verified with the Transmembrane Helices Hidden Markov Model (TMHMM) 2.0 server (http://www.cbs.dtu.dk/services/TMHMM/) (Krogh et al., 2001). In addition, signal peptides were verified using the SignalP 4.1 Server (http://www.cbs.dtu.dk/services/SignalP/) (Petersen et al., 2011). Predicted tRNA regions were verified with the tRNAscan-SE server (http://lowelab.ucsc.edu/tRNAscan-SE/) (Lowe and Eddy, 1997). Inverted repeat sequences were determined using einverted (http://emboss.bioinformatics.nl/cgi-bin/emboss/einverted) (Rice et al., 2011).

### Determination of nearest relatives

The best hit result obtained following BLASTP with an e-value threshold of 10^−3^ (BLAST+ version 2.2.31 (Camacho et al., 2009) matching of phage proteins against the NCBI virus reference sequence database (11 January 2017 update) was used to determine closest viral relatives. Using a classification system similar to that adopted by PHAST (Zhou et al., 2011), the most common phage name (name of the phage with the highest number of proteins most similar to those in the subject phage sequence) and most common phage number (the number of phages with the highest number of proteins most similar to those in the subject phage sequence) were determined for each phage. The bacterial hosts and environmental sources of the most closely related phages were determined using the Virus-host database (Mihara et al., 2016) and manual checks of original publications.

### Phylogenetic analysis and genomic alignment

Phylogenetic analysis was conducted on the basis of the amino acid sequence of phage terminase large subunit genes (*TerL*) of the phages classified within the family Siphoviridae (ϕBrb01, ϕBrb02, and ϕSb01) and head-tail connector proteins for phages classified within the family Podoviridae (ϕRa01 and ϕRa02). Phylogenetic trees for respective phage genes were generated according to host genera (*Bacteroides, Streptococcus*, and *Ruminococcus*) using MEGA7 (Kumar et al., 2016) with ClustalW (Thompson et al., 1994) used for the initial amino acid sequence alignment. The model employed for each phylogenetic analysis was chosen following a model test incorporating a minimum of 56 amino acid substitution models, with the model with the lowest bayesian information criterion (BIC) score used for the final maximum likelihood phylogenetic analysis. The genetic distance presented on each phylogenetic tree was determined on the basis of 1,000 replicate trees (bootstraps). Phylogenetic analysis of the ϕBrb01 and ϕBrb02 *TerL* genes also included eight additional *TerL* genes of integrated prophage elements identified within the genomes of seven herbivore-gut associated *Bacteroides* isolates (Hungate 1000 project, JGI proposal ID: 612). Phylogenetic analysis of the ϕRa02 and ϕRa04 head-tail connector genes included 33 head-tail connector protein sequences obtained from the NCBI reference sequence protein database using the search terms head-tail connector and head-tail joining and an additional seven representative head-tail connector protein sequences from Podoviridae phages infecting hosts classified within the Firmicute genera *Staphylococcus, Streptococcus*, and *Clostridium*. Genomic plots were generated using Geneious R9 (Kearse et al., 2012) and alignments to identify homologous protein regions undertaken in Geneious R9 using Mauve (Darling et al., 2010) and LASTZ (Harris, 2007).

### Analysis of bacterial host genomes: CRISPR analysis, identification of restriction: modification systems, and prophage detection

Host genomes (described in Table 1) included *Bacteroides* sp. AR20 (IMG ID 2596583541, 61 scaffolds), *R. albus* AR67 (IMG ID 2593339152, 103 scaffolds), and *S. equinus* strains 2B (IMG ID 2561511223, 9 scaffolds) Sb04 (IMG IDs 2651870306, 21 scaffolds) and Sb17 (IMG ID 2654588136, 13 scaffolds). The genome annotations from IMG (Markowitz et al., 2012) were used for the detection of genes associated with phage defense systems.

Host bacteria genomes were screened for integrated prophage elements using PHASTer (Arndt et al., 2016) and manual checking of host genome annotations from IMG. Predicted prophage elements were annotated with Prokka (Seemann, 2014) and BLASTP using the virus reference sequence database (January 2017 update). Closest phage relatives were determined using methods described above (section Determination of Nearest Relatives).

CRISPR associated Cas proteins were detected from the IMG genome annotation. CRISPR elements including direct repeats and spacer regions were identified using Prokka and CRISPRFinder (Grissa et al., 2007). Identified direct repeats and spacers were assessed for homology to known direct repeat regions using BLAST+ (version 2.2.31) BLASTN search against the NCBI nr database (January 2017 update) with 10^−3^ e-value threshold. Genome annotations from IMG were used for the detection of genes associated with restriction modification systems using custom word searches and on the basis of homology to restriction-modification system-associated proteins listed in the REBASE proteins database (28th October 2016 download, Roberts et al., 2015), using BLASTP (BLAST+ version 2.2.31), 10^−3^ e-value threshold.

### Genome accession numbers

Whole genome sequence data for phages ϕBrb01, ϕBrb02, ϕRa02, ϕRa04, and ϕSb01 have the JGI genome portal project ID numbers 1035879, 1035881, 1035884, 1035887, and 1035872 respectively. Prophage elements identified during host genome analysis and designated ϕSb2Bpro1 and ϕBrbAR29pro1, were derived from the genome assemblies IMG ID 2561511223 (T517DRAFT_scaffold00001.1, nt 367,615–409,981) and IMG ID 2593339260 (IE59DRAFT_scaffold00020.20, nt 1 to 35,574) respectively.

## Results and discussion

Each phage had a double-stranded DNA genome and their general features are presented in Table 2. Sequencing of phages was completed with a minimum of 380 x sequence coverage and sequence assembly resulted in all phage genomes being represented by a single contig and scaffold. All of the phage sequences were of a similar length to those predicted in the original isolation studies, where genome lengths were estimated on the basis of restriction enzyme digestion patterns (Table 1). The G+C content (%) varied, with the two phages infecting *Bacteroides* sp. AR20, ϕBrb01, and ϕBrb02, having higher GC content than the other phages. All phages had non-coding sequences associated with the complete genomes, and encoded for overlapping reading frames (Table 3). The majority of predicted open reading frames started with ATG, with exception being a single ORF of ϕBrb02 beginning with GTG and two ORFs of ϕSb01 beginning with GTG and a further two ORFs beginning with TTG. No complete tRNA elements were found in any of the lytic phage genomes examined and signal peptides were only identified in the genomes of ϕBrb01 and ϕBrb02.

**Table 2:** Description of phage genomes and nearest relatives including the most common phage name.

**Phage**	**Assembled genome length (kb)**	**G + C content (%)**	**ORFs (no.)**	Most common phage name (number of best hit homologous proteins)^*^	Additional common phage names (number of best hit homologous proteins >1)^**^
ϕBrb01	33.602	45.5	46	*Croceibacter* phage P2559Y NC_023614.1 (4)	*Bacteroides* phage B40-8 NC_011222.1 (2)
ϕBrb02	34.687	45.7	48	*Croceibacter* phage P2559Y NC_023614.1 (4)	*Bacteroides* phage B40-8 NC_011222.1 (1)
					*Bacteroides* phage B124-14 NC_016770.1 (1)
ϕRa02	12.985	35.6	16	n.d.	*Bacillus* phage MG-B1 NC_021336.1 (1)
					*Clostridium* phage phiCPV4 NC_018083.1 (1)
					*Bacillus* virus B103 NC_004165.1 (1)
ϕRa04	12.977	35.3	15	n.d.	*Bacillus* phage MG-B1 NC_021336.1 (1)
					*Clostridium* phage phiCPV4 NC_018083.1 (1)
					*Bacillus* virus B103 NC_004165.1 (1)
ϕSb01	33.595	37.2	46	*Streptococcus* phage 315.5 NC_004588 (10)	*Streptococcus* phage 20617 NC_023503 (3)
					*Streptococcus* phage Str-PAP-1 NC_028666.1 (2)
					*Streptococcus* phage TP-778L NC_022776.1 (2)
					*Streptococcus* phage T12 NC_028700.1 (2)

* *Phage names and NCBI virus reference sequence IDs are listed except where the most common phage name was not determined (n.d.) as the number of best hit homologous proteins did not exceed more than one hit for any one phage*.

** *For some phages the number of best hit homologous proteins did not exceed more than one hit for any one phage. The phage names for all ORF best hits are included for ϕRa02 and ϕRa04 and phage names for hits from the same host genus (Bacteroides) are reported for ϕBrb02*.

**Table 3:** Number of tRNA, signal peptides, and transmembrane (TM) proteins identified in phage genomes.

**Phage**	**Overlapping reading frames**	**tRNA**	**Signal peptides**	**Proteins with TM helices**	**Proteins with >2 TM helices**
ϕBrb01	18	neg	2	6	Holin protein
ϕBrb02	18	neg	1	5	Holin protein
ϕRa02	7	neg	0	1	–
ϕRa04	7	neg	0	1	–
ϕSb01	21	neg	0	2	Tail tape measure protein

### Genome organization and phylogeny of *Bacteroides* phages ϕBrb01 and ϕBrb02

**Figure 1:**
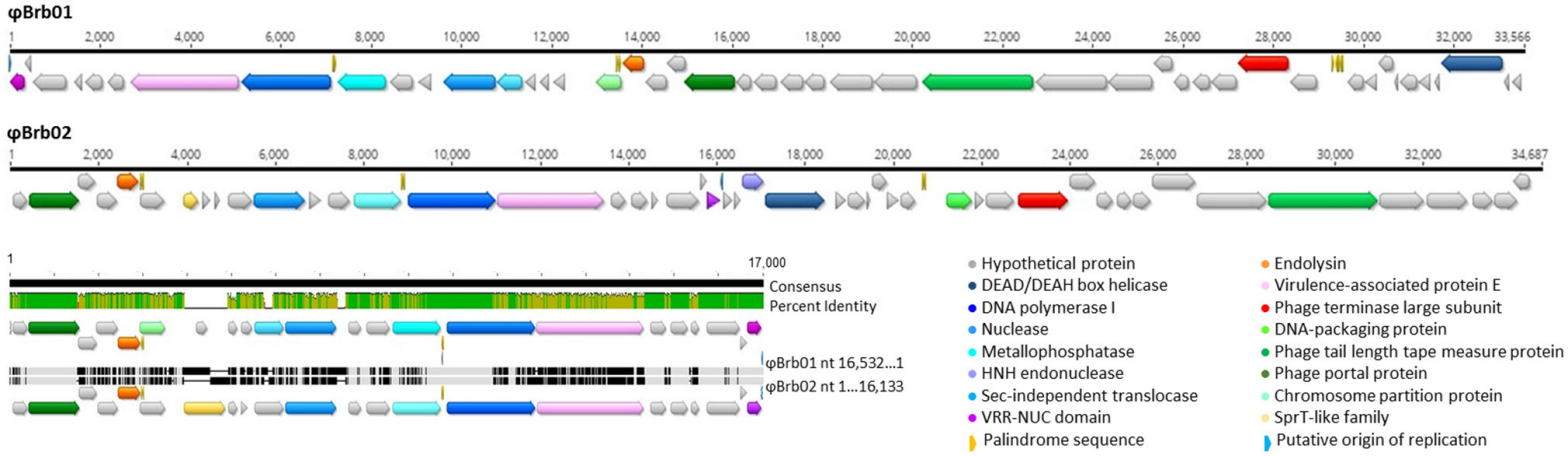
Linear genome arrangement of the *Bacteroides* phages ϕBrb01 and ϕBrb02 showing predicted ORFs and alignment of the most highly homologous region. Predicted ORFs are depicted with arrows colored according to putative gene product names. The alignment was constructed using Mauve (Darling et al., 2010) and includes a consensus sequence (14,790 identical sites) and corresponding percentage identity graph (• 100%, • >30%, • <30%) with differences in the linear alignment of nucleotide sequences indicated with vertical shading.

The two phages infecting the *Bacteroides* sp. strain AR20, ϕBrb01, and ϕBrb02 were previously shown to be of similar genome length and morphology (Table 1), and were isolated from the same source material (Klieve et al., 1991). The genome of ϕBrb02 encoded 48 ORF and was slightly longer in nt sequence length than ϕBrb01 which encoded only 46 ORF (Table 2). Alignment of the phage nt sequences indicated a 40.6% pairwise identity (14,301 identical sites, 1,146 nt gaps). While it had been reported that both of these phage genomes were circularly permuted and that terminal redundancy of the genome occurred during phage particle packaging (Klieve et al., 1991), only the ϕBrb01 genome sequence appeared to have a short (15 nt) overlapping region of terminal redundancy, adjacent to a 24 nt polyA region. This overlapping region was designated as a putative origin of replication, with an identical 15 nt sequence also identified within the ϕBrb02 genome, toward the middle of the genome sequence (Figure 1). Signal peptides and proteins with predicted transmembrane (TM) helices were identified in the genomes of ϕBrb01 and ϕBrb02, indicating that several of the proteins produced by these phages may undergo cleavage events and interact with the host bacterial cell membrane.

Both of the *Bacteroides* phage genomes showed little similarity in overall genome sequence to the previously sequenced human gut-specific lytic phages of *B. fragilis*, B40-8 (Puig and Gironés, 1999) and B124-14 (Ogilvie et al., 2012) and showed greater genetic homology to the *Croceibacter* phage P2559Y (Kang et al., 2016) (Table 2). Only two genes found in the lytic *B. fragilis* phages, encoding for the phage tail length tape measure protein and a hypothetical protein preceding the endolysin protein, were related to the genes present in the ϕBrb01 and ϕBrb02 genomes.

This relative lack of genetic homology to any known viruses meant that for many of the open reading frames identified with the ϕBrb01 and ϕBrb02 genome sequences, gene function could not be predicted with confidence, particularly for expected phage structural proteins (phage tail and capsid proteins). Both ϕBrb01 and ϕBrb02 were isolated on the basis of their lytic ability, as evidenced by the formation of viral plaques within confluent lawns of the AR20 host strain, and no genes for phage integration and maintenance of lysogeny were identified within these phage genomes.

The ϕBrb01 and ϕBrb02 genomes showed some sequence homology to prophage-related gene sequences found within other *Bacteroides* strains (Table 4), including a prophage element found within a bacterial genome assembly of *Bacteroides* sp. AR29. This strain was isolated from ovine rumen fluid in the same laboratory as *Bacteroides* sp. AR20 and had previously been shown to harbor a prophage element which could be chemically induced to form intact phage particles (Klieve et al., 1989). The complete genome for this inducible prophage has been reported (Seet, 2005) although the corresponding prophage sequence obtained from the AR29 genome assembly (GenBank: FOBY01000020.1; IE59DRAFT_scaffold00020.20) was used in the current study (**Figure 7**). Alignment of this scaffold to the lytic *Bacteroides* phage genomes showed extensive homology to 13 genes including those involved in DNA replication and phage particle packaging (for example, the phage terminase large subunit, portal and tail length tape measure proteins). There were also phage proteins in the ϕBrb01 and ϕBrb02 genomes related to prophage elements within other species within the genus *Bacteroides* (Table 4) as well as Firmicutes such as *Bacillus* (*B. licheniformis; B. subtilis*) and *Clostridium* (*C. botulinum*).

**Table 4:** Bacteria with prophage-associated genes homologous to the five lytic phages (ϕBrb01, ϕBrb02, ϕRa02, ϕRa04, and ϕSb01).

**Phage**	Bacteria with homologous proteins (number of homologous phage proteins^*^)
ϕBrb01	*Bacteroides* sp. HMSC067B03 (14); *Bacteroides* sp. AR29 (13); *B. faecichinchillae* (10); *B. fragilis* (8); uncultured *Clostridium* sp. (8)
ϕBrb02	*Bacteroides* sp. AR29 (13); *Bacteroides* sp. HMSC067B03 (13); *B. faecichinchillae* (10); *B. fragilis* (8); uncultured *Clostridium* sp. (8)
ϕRa02	*Flavobacterium* sp. Fl (6)^**^; *Terriglobus* sp. TAA 43 (3)^**^; Candidatus *Moranbacteria* GW2011_GWF2_35_39 (2); *Eggerthella* sp. CAG:1427 (2); *Lucilia cuprina* (2)
ϕRa04	*Flavobacterium* sp. Fl (7)^**^; *Terriglobus* sp. TAA 43 (3)^**^; Candidatus *Moranbacteria* GW2011_GWF2_35_39 (2); *Eggerthella* sp. CAG:1427 (2); *Lucilia cuprina* (2)
ϕSb01	*Streptococcus parauberis* (28); *S. agalactiae* (26); *S. pyogenes* (26); *S. dysgalactiae* (24); *S. iniae* SF1 (21)

*The bacterial names are listed for the top five most-related bacteria only*.

* *Number of respective lytic phage proteins with homology to proteins of listed bacteria [number based on results of BLASTp analysis with the NCBI nr protein database (January 2017 update) with e-value threshold of 10^−3^]*.

** *Possible false positive result*.

**Figure 2:**
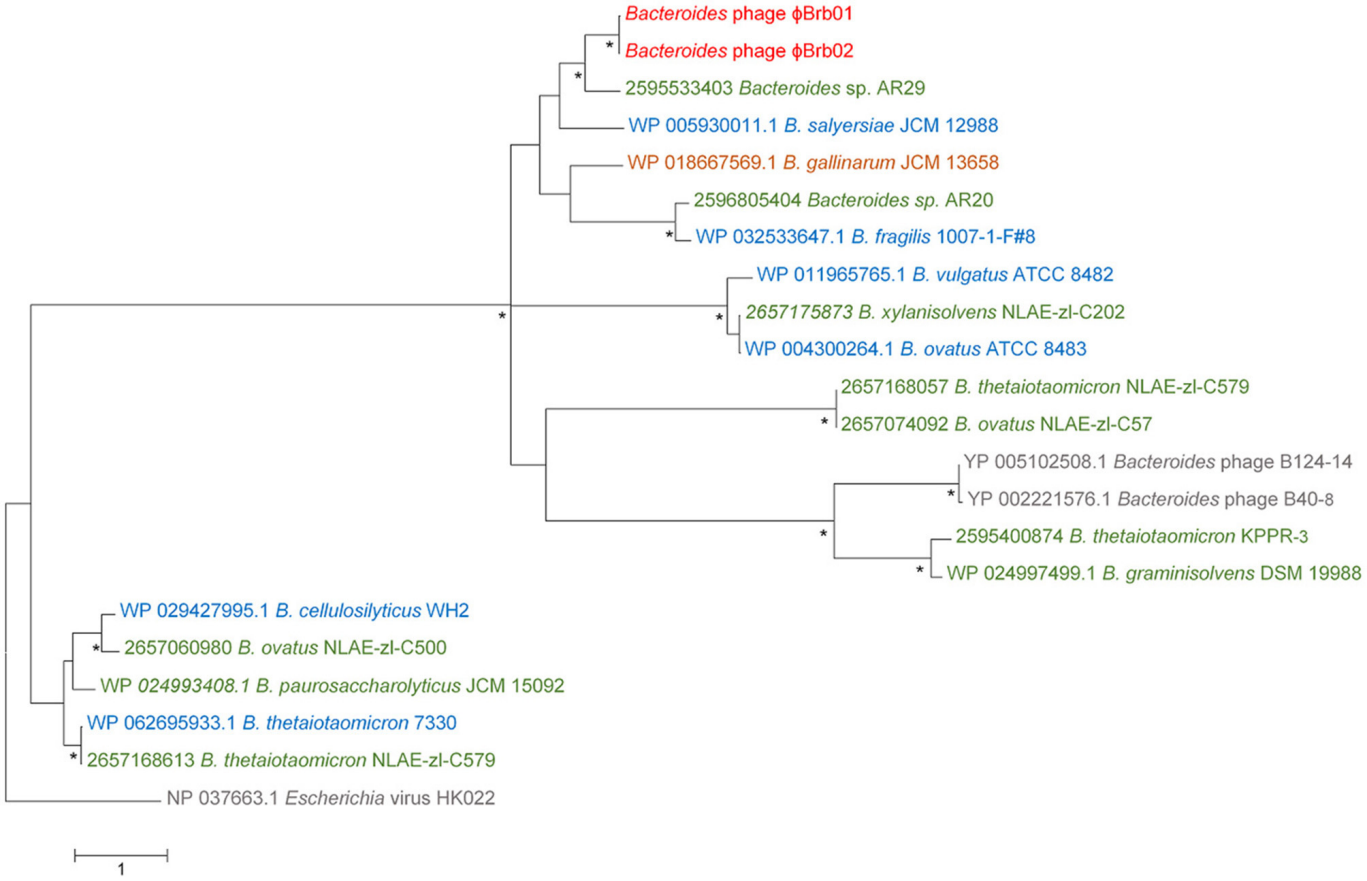
Maximum likelihood phylogeny of the phage terminase large subunit (*Ter*L) proteins of the *Bacteroides* phages ϕBrb01 and ϕBrb02, the virus reference phages B124-14 and B40-8 and *Ter*L protein sequences obtained from the bacterial genome sequences of 17 *Bacteroides* isolates, including 10 sourced from ruminants. The tree with the highest log likelihood (−18,869.80) based on the Le Gascuel model (Le and Gascuel, 2008) and a gamma distribution with five categories is shown. This tree used a total of 1,094 amino acid positions and is drawn to scale, with branch lengths measured in the number of substitutions per site and is rooted with the TerL protein sequence of the *Escherichia* virus HK022. Bootstrap supports greater than 85%, based on 1,000 replicates, are indicated (^*^). The TerL proteins of the *Bacteroides* phages ϕBrb01 and ϕBrb02 are colored red (•). For all other *Ter*L protein sequences, the phage or bacterial genome names and accession numbers are detailed and colored according to the environmental isolation source, including • ruminant origin (rumen fluid, feces and bovine waste); • sewage (waste primarily of human origin); • human origin (gut and patient samples, excluding sewage); and • other gut material (chicken gut).

Phylogeny was also determined on the basis of the Siphoviridae terminase large subunit gene *TerL*, a hallmark gene for phages the order Caudovirales (Iranzo et al., 2016). This analysis incorporated *TerL* genes identified within the bacterial genome assemblies of *Bacteroides* strains isolated from ruminant gut and waste material and phage reference sequences. There was no clear distinction between either the isolation source or *Bacteroides* host-specific clustering of the *TerL* genes, however the three phages derived from ovine rumen isolates did show the greatest *TerL* protein sequence homology (Figure 2).

### Genome organization and phylogeny of *Ruminococcus* phages ϕRa02 and ϕRa04

**Figure 3:**
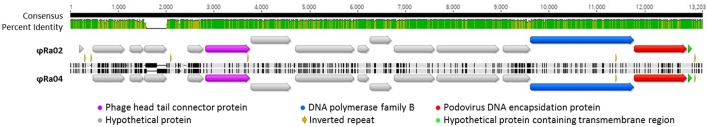
Genomes and alignment of the *Ruminococcus* phages ϕRa02 and ϕRa04. Predicted ORFs are depicted with arrows and putative gene products colored accordingly. The alignment was constructed using Mauve (Darling et al., 2010) and includes a consensus sequence and corresponding percentage identity graph (• 100%, • >30%, • <30%) with differences in the linear alignment of nucleotide sequences indicated with vertical shading.

The two phages infecting *R. albus* strain AR67 were previously classified within the family Podoviridae (Klieve et al., 2004). The genomes of these phages were found to be genetically very similar (95.68% nt sequence homology) with identical genomic arrangement (Figure 3). The main difference between the two phages occurred at the start of the sequence, with ϕRa02 having an additional short predicted ORF (35 amino acid sequences in length) which was not found in the ϕRa04 sequence. The ϕRa02 sequence also encoded an additional five amino acids in the 4th predicted ORF with this protein being predicted to confer some outside transmembrane potential as determined by TMHMM analysis, thus potentially being able to interact with the bacterial cell membrane. The ϕRa02 sequence also included three inverted repeats, whereas the ϕRa04 sequence had only one, which exactly matched one of the inverted repeat sequences detected in ϕRa02.

The lack of any highly-related phage genomes limited the extent to which these genomes could be annotated and the most common phage name could not be determined (Table 2). The few phage genes for which identity could be predicted were all related to genes previously shown to be highly conserved in Podovirus genomes (Iranzo et al., 2016), including the head-tail connector protein, DNA polymerase B and Podovirus encapsidation proteins.

**Figure 4:**
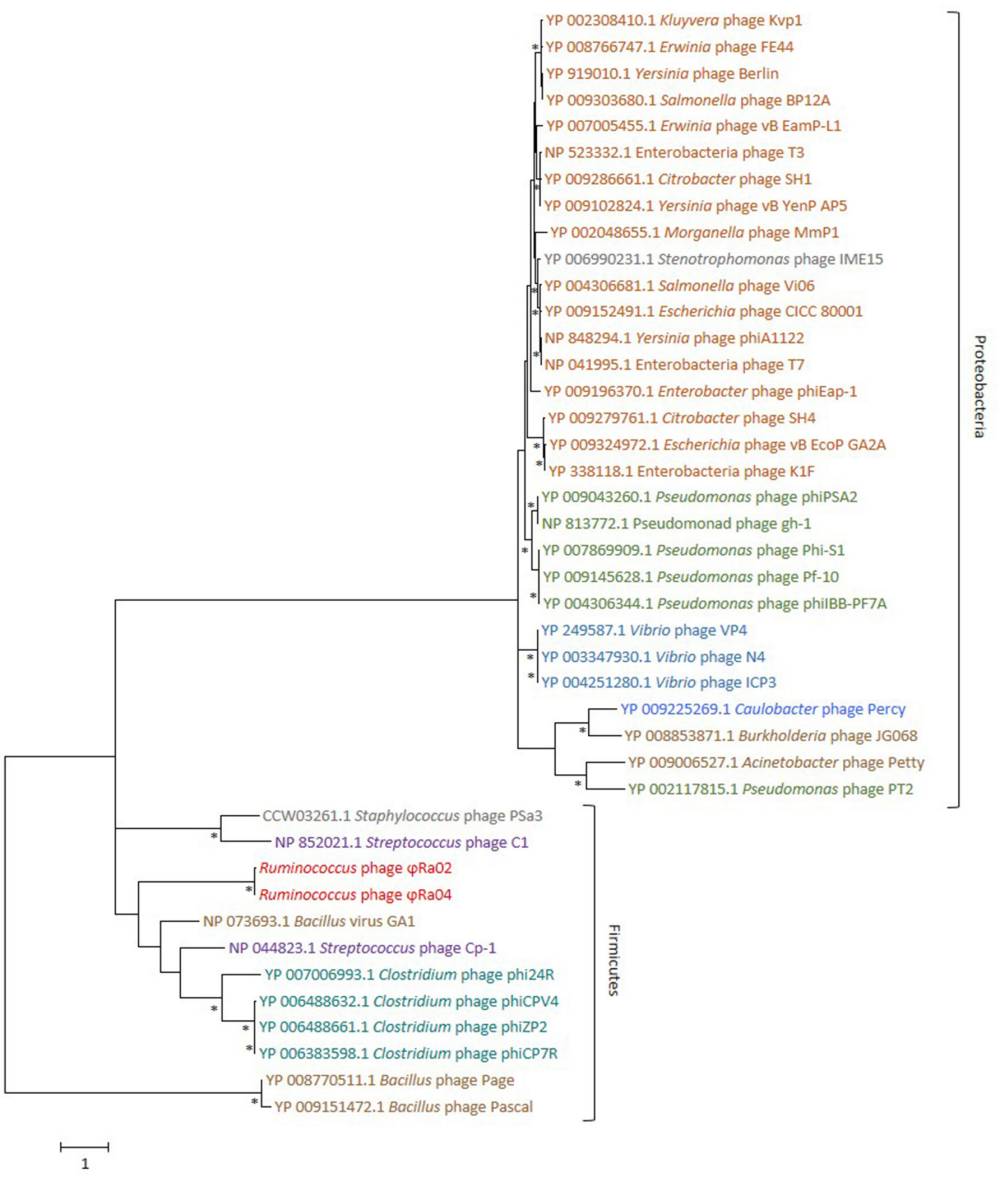
Maximum Likelihood phylogeny of the Podovirus head-tail connector protein sequences of the *Ruminococcus* phages ϕRa02 and ϕRa04 and 40 Podoviruses. The tree with the highest log likelihood (−19562.46) using the Le Gascuel model (Le and Gascuel, 2008) and a gamma distribution with five categories is shown. The rate variation model allowed for some sites to be evolutionarily invariable (0.02% sites). This tree used a total of 707 amino acid positions and is drawn to scale, with branch lengths measured in the number of substitutions per site. The tree is rooted with head-tail connector protein sequences of two *Bacillus megaterium* phages and bootstrap supports greater than 85%, based on 1,000 replicates, are indicated (^*^). Major phyla are bracketed and phage names and accession numbers are colored according to the phage-host family (Enterobacteriaceae •), genera (*Pseudomonas* •, *Vibrio* • and *Ruminococcus* •, Clostridium •) or environmental source (soil-associated *Bacillus* and *Acinetobacter* •, human respiratory tract associated *Streptococcus* • and water-associated *Caulobacter* •). Names of phages with hosts classified outside of these taxons or of unknown isolation source (*Stenotrophomonas* and *Staphylococcus*) are colored gray •.

Phylogenetic analysis on the basis of the signature Podovirus gene, the head-tail connector protein (Iranzo et al., 2016), showed that phages infecting hosts of the same or highly related genera for example, phages infecting the Gram negative family Enterobacteriaceae, usually clustered together (Figure 4). In accordance with this, as the phages ϕRa02 and ϕRa04 infect a *Ruminococcus* host classified within the Gram positive bacterial phylum Firmicutes, the head-tail connector proteins of these phages clustered more closely with phages infecting the Firmicutes genera *Bacillus, Clostridium*, and *Streptococcus* (Figure 4). There was however a greater extent of genetic diversity in the head-tail connector genes of the phages infecting bacteria classified within the Gram positive phylum Firmicutes (for example *Bacillus*), compared to the Podoviruses infecting bacterial genera classified within the Gram negative phylum Proteobacteria (for example *Yersinia, Pseudomonas*, and *Vibrio*). The head-tail connector genes of phages ϕRa02 and ϕRa04 were distinctly different to those found in most other previously sequenced Podoviruses, being most like the head-tail connector protein of the phages such as GA1, infecting *B. subtilis* and classified with the Podovirus subfamily, Picovirinae, and Phi29 genus (Gascon et al., 2000).

An additional gene identified within the ϕRa02 and ϕRa04 phage sequences, the Podovirus encapsidation protein or DNA packaging ATPase (Pfam 05894.6) is also characteristic of the Phi29-like viruses (Garvey et al., 1985; Volozhantsev et al., 2012). This gene was flanked in the ϕRa02 and ϕRa04 genomes with a preceding DNA replication protein (DNA polymerase family B) and a terminal protein with predicted N-terminal signal sequence and transmembrane helix, indicating the presence of an N-terminally located transmembrane-spanning domain of 20 amino acids. This genetic arrangement is characteristic of Phi29-like viruses which have been shown to undergo membrane-associated DNA replication (Meijer et al., 2001b). In addition, the ϕRa02 and ϕRa04 genomes are of similar length, <20 kb and encoded approximately 16 ORF, similar to other Phi29-like phages (Meijer et al., 2001a; Redondo et al., 2013).

The ϕRa02 and ϕRa04 genomes were also unlike most prophage sequences associated with bacterial genomes (Table 4). The ϕRa02 and ϕRa04 genomes were however highly homologous (100% nt homology) to genomic scaffolds associated with the genome sequences of two bacteria isolated from aquatic sediment and soil (Eichorst et al., 2007; McTaggart et al., 2015), specifically two short contigs (6,985 and 5,502 nt) of the *Flavobacterium* sp. F1 assembly (NCBI Reference sequence scaffolds NZ_JQJY01000012.1 and NZ_JQJY01000014.1) and a single contig (12,818 nt) in the genome assembly of *Terriglobus* sp. TAA 43 (NCBI Reference sequence NZ_JUGR01000007.1). The lack of any genes relating to phage integration within these scaffolds and the very high nucleotide sequence homology observed, suggests that these findings be treated with caution. The differences in G+C content of these phage-related scaffolds compared to the remainder of the bacterial genome assembly also suggests possible sequence contamination. For example, the *Terriglobus* genome has seven scaffolds, six of which have G+C of 54–58% whereas the phage-related scaffold has G+C of 35%.

### Genome organization and phylogeny of the *Streptococcus* phage ϕsb01

**Figure 5:**
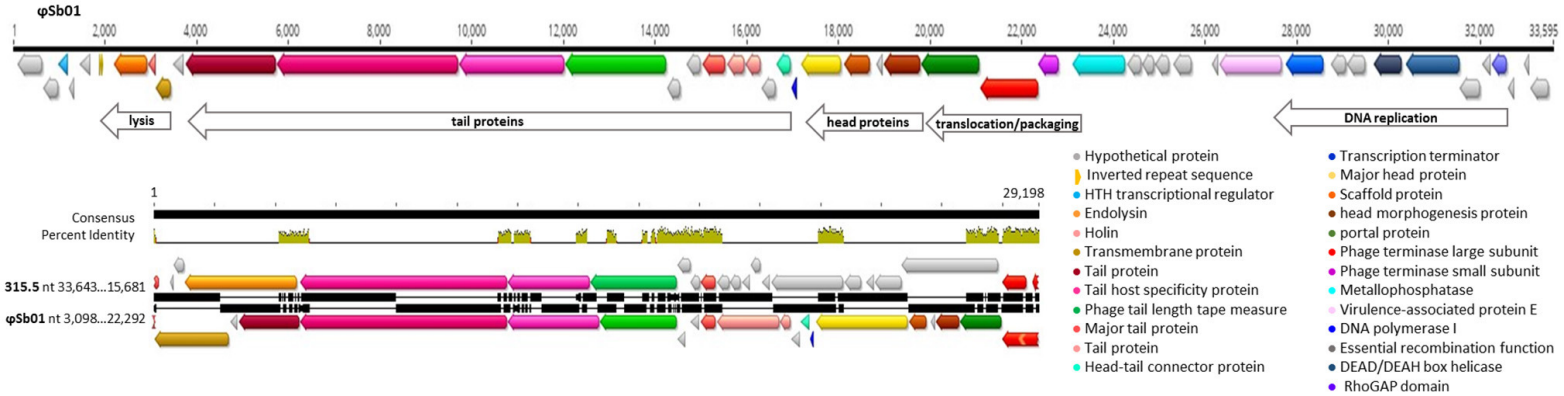
Genome arrangement of the *Streptococcus* phage ϕSb01 showing predicted ORFs and alignment with a homologous region of the *Streptococcus* phage 315.5 (NCBI virus reference sequence NC_004588). Modular groupings of phage genes with similar functional roles are described and predicted ORFs are depicted with arrows colored according to putative gene products. The alignment was constructed using Mauve (Darling et al., 2010) and includes a consensus sequence and corresponding percentage identity graph (• 100%, • >30%, • <30%) with differences in the linear alignment of nucleotide sequences indicated with vertical shading.

The *Streptococcus* phage ϕSb01 was the rumen phage most highly related to previously reported phages, with 34 of the 35 ORFs for which gene function could be assigned, having protein homology to previously characterized *Streptococcus* phage and prophage-associated genes (Tables 2, 4). The only exception was one ORF (essential recombination function protein YP_009216915.1) which was homologous to a protein from the *Clostridium* phage phiCDHM19 (Hargreaves and Clokie, 2015). The ϕSb01 genome was found to be organized into modular groups relating to gene function (Figure 5). For example, genes relating to phage particle structure including tail proteins (major and minor structural tail proteins, tail host-specificity protein, tail length tape measure protein, and head-tail connector proteins) were clustered together. These genes were also adjacent to a transcription regulator, indicating that phage genome replication was regulated and controlled through sequential transcription of phage genes (late and early gene replication).

The nearest phage relative to ϕSb01 on the basis of protein homology was the *S. pyogenes* phage 315.5 (Beres et al., 2002) with a conserved module of 10 genes encoding proteins responsible for phage particle structure and morphogenesis, including tail proteins, a phage:host specificity minor tail fiber protein and phage head and head morphogenesis proteins (Figure 5). The major phage packaging genes (phage portal and terminase genes) were most like the *Streptococcus* phage T12 which is able to infect and form a lysogenic association with group A *Streptococcus* (GAS) strains (Zabriskie, 1964; McShan et al., 1997). When originally isolated from bovine rumen fluid, ϕSb01 particles were shown to replicate and produce clear plaques on lawns of bacterial host (Klieve and Bauchop, 1991) and pure preparations of this phage were subsequently shown to rapidly clear liquid cultures, indicating phage lytic replication. While the genome of this phage did contain several genes involved in gene regulation, no integrase genes were identified, further indicating that this phage was unable to integrate and form a stable, lysogenic associations with its bacterial host.

**Figure 6:**
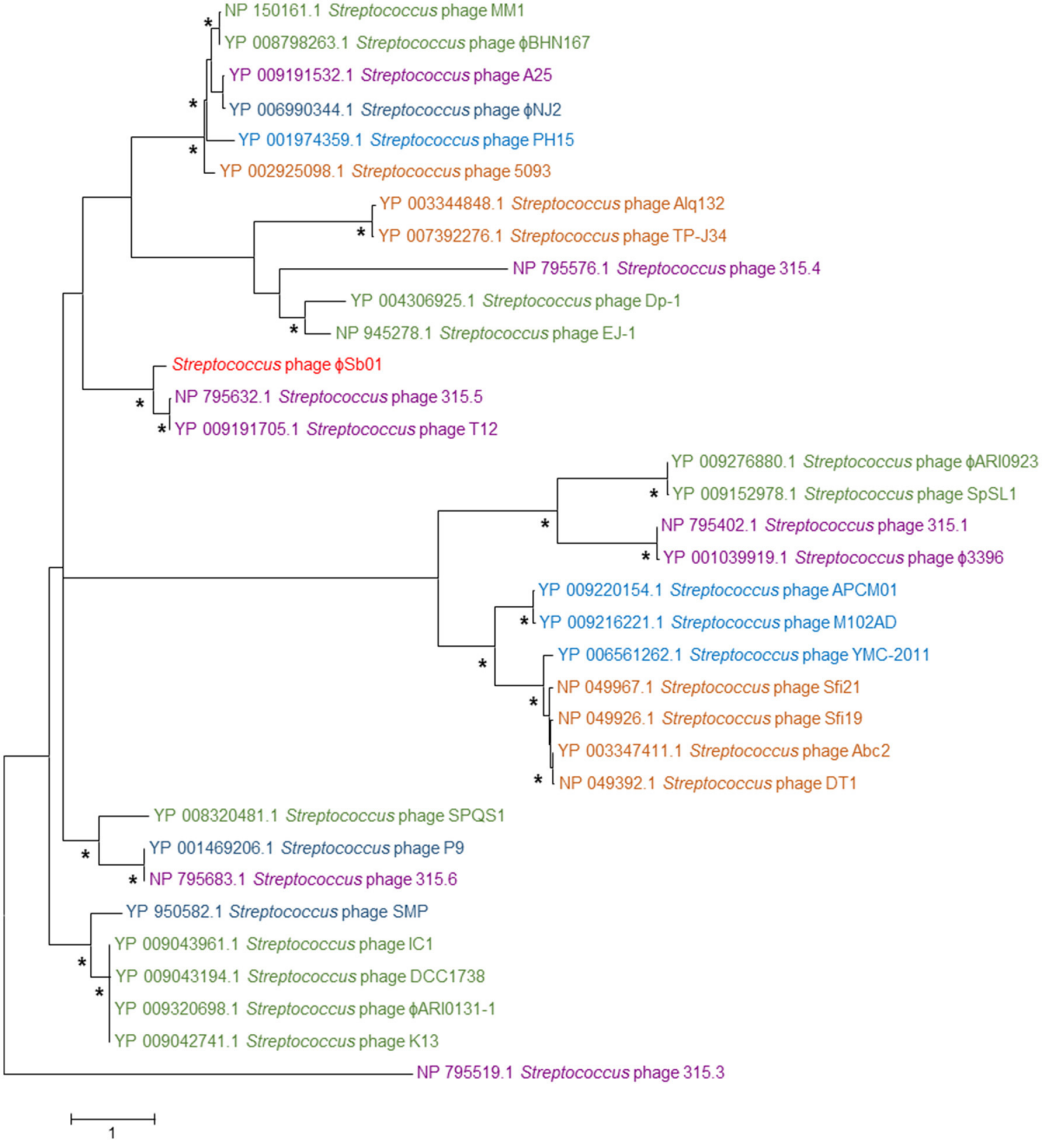
Maximum likelihood phylogeny of the phage terminase large subunit (*Ter*L) proteins of the *Streptococcus* phage ϕSb01 and representative reference phages infecting the genus *Streptococcus*. The tree with the highest log likelihood (−21,218.68) based on the Whelan and Goldman model (Whelan and Goldman, 2001) with frequencies and a gamma distribution with five categories is shown. This tree used a total of 667 amino acid positions and is drawn to scale, with branch lengths measured in the number of substitutions per site and is rooted with the *Ter*L protein sequence of the *Streptococcus* phage 315.3. Bootstrap supports greater than 85%, based on 1,000 replicates, are indicated (^*^). The *Ter*L proteins are colored according to phage host species and environmental isolation source • *S. bovis/equinus* 2B (rumen, gut-associated); • *S. pneumoniae* (human respiratory tract); • *S. pyogenes*, GAS strains and *S. dysgalactiae* subsp. *equisimilis* (human, skin flora, respiratory tract); • *S. suis* and *S. equi* (non-human, mammal respiratory tract); • *S. mutans, S. salivarius*, and *S. gordonii* (human dental and oral); • *S. thermophilus* (dairy fermentations, whey).

Phylogeny on the basis of the terminase large subunit gene of phages known to infect human, animal and environmental isolates of the genus *Streptococcus* (Figure 6), showed that the *TerL* protein of this phage was most similar to the *TerL* genes of phages infecting *S. pyogenes* of human respiratory tract origin.

### Phage:host resistance mechanisms

Five whole genome sequences representing at least one bacterial host strain for the five lytic phages, were examined. Host strains included (1) *Bacteroides* sp. AR20, the Gram negative, anaerobic bacterial host for ϕBrb01 and ϕBrb02 and classified within the Fibrobacteres-Chlorobi-Bacteroidetes superphylum (FCB group), Bacteroidetes phylum; (2) *R. albus* AR67, the Gram positive, anaerobic bacterial host of ϕRa02 and ϕRa04 and classified within the Phylum Firmicutes, order Clostridiales; and (3) three known bacterial hosts of ϕSb01, *S. equinus strains* 2B, Sb04 and Sb17 which are all Gram positive, facultative anaerobes, classified within the Phylum Firmicutes, order Lactobacillales. All bacterial sequences were interrogated to identify genetic factors which may contribute to phage resistance including prophage elements, Clustered Regularly Interspaced Short Palindromic Repeats (CRISPR) and restriction modification (RM) systems. The five lytic phages reported in this study however, must be unaffected by, or be able to counteract, these resistance mechanisms as they can all successfully infect and replicate within these bacteria.

Phage-related genes were found to be present in all the bacterial host genomes examined (Table 5) and were often present as modules of genes resembling integrated prophage-like elements. The prophage-like elements identified in *Bacteroides* sp. AR20, the *S. equinus* strains Sb04 and Sb17 and *R. albus* AR67 incorporated phage integrase and DNA replication genes and were of variable lengths (predicted sequence length ranging between 4.7 and 32 kb). Genes for major structural proteins or phage particle morphogenesis were usually not associated with these prophage regions and given the relatively low number of ORFs identified, these regions most likely represent incomplete remnants of phage or horizontally-transferred genetic elements. Moreover, sequence alignment of these regions with each of the respective lytic phage genomes indicated that the majority of genes found in these incomplete phage-like regions, were not homologous to the genes found in the lytic phages.

**Table 5:** Description of predicted prophage regions identified in the bacterial host strain genomes *Bacteroides* sp. AR20, *R. albus* AR67, and *S. equinus* strains 2B, Sb04, and Sb17.

**Prophage region**	**Region length (bp)**	**G + C content (%)**	**ORFs (no.)**	Most common phage name (number of best hit homologous proteins)^*^	Additional common phage names (number of best hit homologous proteins)^**^
ϕBrbAR20pro1	27,691	38.6	32	n.d.	*Acanthocystis turfacea Chlorella* virus 1 (1); *Cafeteria roenbergensis* virus BV-PW1 (1); *Bathycoccus* sp. RCC1105 virus BpV1 (1); *Mycobacterium* phage Kratio (1); *Streptococcus* virus 9872 (1); *Staphylococcus* phage phiN315 (1); *Bacillus* phage SP-15 (1); Only Syngen Nebraska virus 5 (1); *Staphylococcus* phage phiN315 (1); Cedratvirus A11 (1); Stx2-converting phage 1717 (1); *Acanthamoeba polyphaga* moumouvirus (1); *Enterococcus* phage EF62phi (1)
ϕBrbAR20pro2	29,595	46.7	28	n.d.	*Natrialba* phage PhiCh1 (1); *Gordonia* phage Nyceirae (1); *Bacillus* phage BM5 (1); *Bacillus* phage vB_BanS-Tsamsa (1); *Clostridium* phage c-st (1); *Arthrobacter* phage vB_ArS-ArV2 (1); *Pseudomonas* phage YuA (1); *Ostreococcus mediterraneus* virus 1 (1)
ϕBrbAR20pro3	4,783	40.7	5	*Riemerella* phage RAP44 NC_019490.1 (2)	*Clostridium* phage phiCT9441A (1)
ϕRaAR67pro1	5,340	41.5	9	n.d.	*Clostridium* phage phiCT19406C (1); *Geobacillus* phage GBSV1 (1); *Paenibacillus* phage Vegas (1)
ϕRaAR67pro2	17,507	50	22	n.d.	*Clostridium* phage phiSM101 (1); *Brevibacillus* phage Jenst (1); Deep-sea thermophilic phage D6E (1); *Paramecium bursaria Chlorella* virus FR483(1)
ϕRaAR67pro3	8,051	37.8	8	n.d.	*Bacillus* phage phIS3501 (1); *Thermus* phage P2345 (1); *Brevibacillus* phage Jenst (1); *Enterobacteria* phage fiAA91-ss (1); *Clostridium* phage CDMH1 (1); *Aureococcus anophagefferens* virus (1)
ϕSb2Bpro1	42,367	38.5	52	*Streptococcus* phage P9 NC_009819 (6)	*Streptococcus* phage 315.6 (6); *Bacillus* phage BCJA1c (4); *Enterococcus* phage EFC-1 (2); *Staphylococcus* phage X2 (2); *Streptococcus* phage SM1 (2); *Streptococcus* phage phiARI0468-2 (2)
ϕSb2Bpro2	10,673	38.5	15	n.d.	*Clostridium* phage phi3626 (1); *Clostridium* phage phiMMP02 (1); *Vibrio* phage douglas 12A4 (1); *Mycobacterium* phage Squirty (1); *Acidianus* two-tailed virus (1); *Geobacillus* phage GBSV1 (1); *Acinetobacter* phage Acj9 (1)
ϕSeSb04pro1	16,394	37.1	15	*Bacillus* phage SPBc2 NC_001884.1 (3)	*Streptococcus* phage Dp-1 (2); *Bacillus* virus G (1); *Acanthocystis turfacea Chlorella* virus 1 (1); *Aureococcus anophagefferens* virus (1); *Cellulophaga* phage phiSM (1); *Bacillus* phage BCD7 (1); *Lactococcus* phage bIL311 (1); *Synechococcus* phage S-SSM7(1)
ϕSeSb04pro2	6,961	37.9	9	n.d.	*Streptococcus* phage 315.3 (1); *Aeropyrum pernix* spindle-shaped virus 1 (1); *Escherichia* phage phAPEC8 (1); *Sphingomonas* phage PAU (1); *Enterobacteria* phage phi92 (1); *Streptococcus* phage phiARI0460-1 (1); *Acinetobacter* phage ZZ1(1)
ϕSeSb04pro3	8,194	35.4	9	*Bacillus* virus G NC_023719.1 (2)	*Acanthamoeba polyphaga mimivirus* (1); *Planktothrix* phage PaV-LD (1); *Lactobacillus* phage LfeInf (1); *Staphylococcus* phage phiIPLA-C1C(1)
ϕSeSb17pro1	12,602	33.1	21	*Streptococcus* phage EJ-1 NC_005294.1 (3)	*Lactococcus* phage bIL310 (2); *Streptococcus* phage phi3396 (2); *Lactococcus* phage bIL311 (2); *Streptococcus* phage 315.3 (2)

* *Most common phage names and NCBI Virus reference sequence IDs are listed except where the most common phage name was not determined (n.d.) as the number of best hit homologous proteins did not exceed more than one hit for any one phage*.

** *Additional common names listed include those where the number of best hit homologous proteins >1. Where the number of best hit homologous proteins never exceeded more than one phage name, all phage names corresponding to each ORF best hit homologous protein match are listed*.

**Figure 7:**
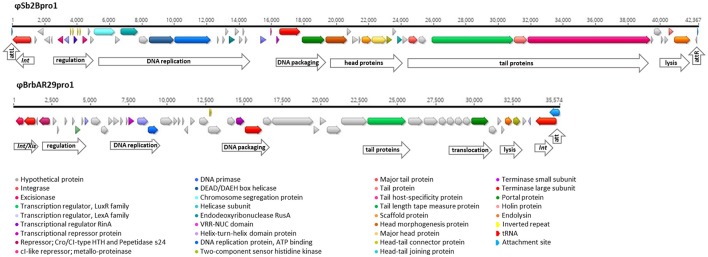
Genome arrangement of prophage regions identified in the bacterial genome sequences of *Streptococcus equinus* strain 2B (ϕSb2Bpro1) and *Bacteroides* sp. AR29 (ϕBrbAR29pro1). Modular groupings of phage genes with similar functional roles are described and predicted ORFs are depicted with arrows colored according to putative gene products. Predicted phage attachment sites (att), inverted repeat sequences and a tRNA element are also depicted.

The exception to this was the *TerL* gene of ϕSb01 which was similar (69% pairwise nt identity) to the *TerL* gene present within the relatively long (42.3 kb) prophage element identified within *S. equinus 2B*. This prophage element, designated ϕSb2Bpro1 was the only host-related element identified in the study which contained a potentially complete complement of phage genes. Prophage ϕSb2Bpro1 encoded 52 ORFs and included genes with homology to both structural and non-structural phage proteins arranged in a modular sequence, interspaced by regulatory control genes (Figure 7). In contrast to the lytic phage ϕSb01, the genome of ϕSb2Bpro1 incorporated the key genes required for phage integration and maintaining lysogeny through regulation of gene expression.

CRISPR associated proteins (*Cas* proteins) were found to be present in all the bacterial strains examined (Table 6), with the exception of *R. albus* AR67, which had several relatively short CRISPR direct repeat (DR) regions present in multiple scaffolds of the AR67 genome assembly. This may be attributed to the relatively incomplete nature of this genome assembly (103 scaffolds), which may have hampered the detection of CRISPR-*Cas* regions with a full complement of genes. Interestingly, of all the CRISPR regions identified, none showed any spacer sequence homology to the lytic phage genome sequences reported in this study.

**Table 6:** CRISPR/*Cas* elements identified in bacterial host genome sequences.

**Bacterial host strain**	*Cas* proteins^*^	DR (no. repeats; repeat length), nt co-ordinates^**^	DR homology (10^−3^ e-value threshold)^***^	**Spacers (no.)**	Spacer sequence homology (10^−3^ e-value threshold)^****^
*Bacteriodes* sp. AR20	None	DR1 (4, 23 nt) contig 52.52 nt 609–778	No homology	3	*R. albus* plasmid pAR67 (D88665.1)
*R. albus* AR67	None	DR1 (4; 24 nt) contig1.1 nt 261160–261418	No homology	3	No homology
		DR2 (4, 24 nt) contig 1.1 nt 261550–261807	No homology	3	No homology
		DR3 (4, 24 nt) contig 14.14 nt 9638–9973	No homology	4	No homology
		DR4 (12, 23 nt) contig 31.31 nt 1118–1998	No homology	11	No homology
		DR5 (4, 24) contig 44.44 nt 14202–14460	No homology	3	No homology
*S. equinus* 2B	*Cas*2, *Cas*1, *Csn*1	None			
*S. equinus* Sb04	*Csn*1, *Cas*1, *Cas*2, *Csn*2, *Cas Csm*6	DR1 (21; 36) contig 101.101 nt 253157–254514	*S. thermophilus* strain ND07 (CP016394.1); *S. thermophilus* strain KLDS (CP016877.1); *S. thermophilus* strain KLDS SM (CP016026.1)	20	No homology
*S. equinus* Sb17	*Cas Csm*6	None			

* *CRISPR associated (Cas) proteins from IMG genome annotation*;

** DR with >3 repeats;

*** *DR homology, top 3 best hits results, BLASTn against the NCBI nr database (January 2017 update), e-value threshold of 10^−3^*.

**** *Spacer sequence homology top 3 best hits results, BLASTn against the NCBI nr database (January 2017 update), e-value threshold of 10^−3^*.

The two *Streptococcus* strains, 2B and Sb17 had genes for *Cas* proteins however no CRISPR direct repeats (DR) were either associated with these genes or detected elsewhere within the genome assemblies. The *Bacteroides* sp. AR20 genome contained a CRISPR incorporating three spacer regions however there were no *Cas* proteins identified within this genome. Two of the AR20 CRISPR spacer regions were homologous (100% homology over the complete 32 nt spacer region) to a plasmid replication protein previously identified in *R. albus* AR67 (Ohara et al., 1998). The most complete CRISPR element was identified in the genome of *S. equinus* Sb04, with the DR sequences being highly related to those found in other *Streptococcus* (Table 6). This CRISPR region had all the elements of a type II-A CRISPR-*Cas* system with a genomic arrangement similar to those previously observed in *S. thermophilus* and *S. pyogenes* (Barrangou, 2013; Shmakov et al., 2017) incorporating the consecutive proteins, *Csn*1, *Cas*1, *Cas*2, and *Csn*2 followed by the CRISPR repeat region. The *Cas* proteins and two of the DR in this CRISPR element also showed extensive sequence homology to a complete CRISPR-*Cas* system present in the *Streptococcus infantarius* subsp. *infantarius* strain CJ18 (Jans et al., 2013) with > 94% nt homology to *Cas* and *Csn* proteins and 100% nt homology to two of the DRs of 35 and 36 nt in length.

Restriction enzyme and DNA methylase sequences were identified in all of the bacterial host genomes (Table 7) however the fragmented nature of the AR67 genome assembly made it difficult to identify the modules of consecutive genes characteristic of some restriction-modification systems. The most complete Type I RM systems were identified in the genomes of *S. equinus* Sb04 and *S. equinus* Sb17, recognizable on the basis of sequential genes representing multi-subunit systems of Type I restriction enzyme specificity (S) subunits, Type 1 methyltransferase (M) and Type I restriction (R) enzymes. These enzymes can act in combination to recognize bi-partite motifs and cleave at large distances from their binding site (Blow et al., 2016).

**Table 7:** Number of bacterial host genes homologous to genes associated with restriction-modification systems (10^−3^ threshold BLASTp search against the REBASE protein database).

Restriction-modification system related genes (REBASE classifications^*^)	***Bacteroides* sp. AR20**	***R. albus* AR67**	***S. equinus* 2B**	***S. equinus* Sb04**	***S. equinus* Sb17**
Putative control protein	5	11	7	5	9
Putative homing endonuclease	8	6	8	8	8
Homing endonuclease		2			
Putative orphan methyltransferase	1	1	1	1	1
Putative Type I restriction enzyme	3	7	7	6	6
Type I specificity subunit	3			9	
Putative Type I specificity subunit	15	13	9	9	11
Type I methyltransferase	8	7	9	8	8
Putative Type I methyltransferase	10	10	6	9	8
Putative Type II specificity subunit	1	1	1	1	1
Type II methyltransferase					1
Putative Type II methyltransferase	23	44	22	20	19
Putativetype II nicking endonuclease	2	2	3	2	2
Putative Type II restriction enzyme	41	30	12	13	17
putative Type II helicase domain protein		1			
Type IIG restriction enzyme/methyltransferase			1		1
Putative Type IIG restriction enzyme/methyltransferase	43	49	12	12	11
Putative Type III restriction enzyme	2				
Type III methyltransferase				1	
Putative Type III methyltransferase		1			
Putative Type IV methyl-directed restriction enzyme	32	33	26	26	23

* *Restriction modification type classifications from the REBASE protein database, October 2016 (Roberts et al., 2015) including biochemically characterized proteins and predicted proteins (putative)*.

Intact Type I RM systems were not identified in the other rumen bacterial host genomes, with the *Bacteroides* sp. AR20 and *R. albus* AR67 genomes instead containing multiple copies of Type II RM systems, Type IIG restriction/methyltransferase genes and Type IV methyl-directed restriction enzymes. These RM systems are simpler in structure with the Type II RM systems comprising restriction endonuclease and DNA methyltransferase enzymes which are expected to show identical DNA-binding specificity (Pingoud et al., 2014). The Type IIG enzymes which contain both DNA restriction and methylation activities (Blow et al., 2016), were more abundant and diverse in the *Bacteroides* and *Ruminococcus* genomes than the three *Streptococcus* genomes. The *Streptococcus* genomes Sb04 and Sb17 both encoded multiple copies of the putative Type IIG restriction enzyme/methyltransferase MboSP38ORF5300 (from *Mycobacterium bovis* strain SP38, Genbank CP015773). *S. equinus* 2B also encoded a second putative Type IIG restriction enzyme/methyltransferase, Sin18ORF1583, from *S. infantarius* subsp. *infantarius* CJ18 (AEZ62887.1). In contrast *R. albus* AR67 encoded multiple copies of seven different putative Type IIG restriction enzyme/methyltransferases (Ral7ORF3617, MboSP38ORF5300, UbaMSORFI6, SstMg1ORFD; AviDJORF22890, HauORF2540, and Ral7ORF3270) and *Bacteroides* sp. AR20 encoded multiple copies of 11 different putative Type IIG restriction enzyme/methyltransferases (Mpu984ORF12500, OrhH06ORF7025, Bxy1bORF28720, Bxy1bORF28730, PkoX141TORF16370, DxiR13ORFI, MspMAB1ORF830, Htr1232ORFE, HmgGUTORFU2, AspNJ1ORF21265, MboSP38ORF5300). The Type IV RM systems, distinguished by their ability to cut modified DNA without relying on an associated methyltransferase component (Loenen and Raleigh, 2014), were also more predominant in the AR20 and AR67 genomes than the *Streptococcus* genomes.

## Conclusion

This study reports the first full genome sequences obtained for lytic phages that infect bacteria isolated from the rumen. These phages were sourced directly from the rumen and ruminant-housing and waste-water environments and infect Firmicutes (*Ruminococcus* and *Streptococcus*) and Bacteroidetes (*Bacteroides*). The genome sequences were novel in their genetic composition and while some more conserved phage genes could be identified, for example, DNA polymerase, head-tail connector proteins, and phage DNA packaging genes (terminase large subunit genes), the majority of phage genes encoded by these lytic phages could not be assigned functional roles on the basis of sequence homology. This was most apparent for the phages ϕRa02 and ϕRa04 infecting *R. albus* AR67, where only three genes could be annotated with confidence. Drawing on these few genes for phylogenetic analysis and the prediction of proteins with transmembrane potential, enabled these tailed phages to be further classified within the Podovirus subfamily, Picovirinae and the Phi29 genus.

The phages infecting *Bacteroides* sp. AR20, ϕBrb01, and ϕBrb02 were also genetically novel, showing little similarity to the two previously sequenced lytic phages infecting the *Bacteroides* genera, *B. fragilis*, B40-8 (Puig and Gironés, 1999) and B124-14 (Ogilvie et al., 2012). Despite the dominance and importance of the *Bacteroides* taxa in the human gut (Ndeh et al., 2017) and a history of lytic phages infecting the genera Bacteroides being employed for the purposes of tracking fecal contamination of waterways (Puig et al., 1999), very few prophage elements or lytic phages infecting the genus *Bacteroides* have been genetically described. Within the ϕBrb01 and ϕBrb02 genomes, conserved phage genes including those involved in phage DNA replication (DNA polymerase I and helicases), particle packaging and translocation (terminase large subunit and portal proteins) and host cell lysis (phage endolysin) could be identified. Phylogenetic analysis on the basis of the terminase large subunit gene and comparative analysis on the basis of predicted protein sequences, indicated that the ϕBrb01 and ϕBrb02 genomes were most closely related to prophage elements associated with bacterial isolates of *Bacteroides* and an intact prophage present in the genome of an ovine rumen *Bacteroides* isolate, strain AR29. The ϕBrb01 and ϕBrb02 genomes however lacked genes for phage integration and replication control, indicating that they could not establish a stable, lysogenic association with their bacterial host. These genomes however were also quite unique in their genetic composition, containing many genes of unknown function. Anticipated structural phage genes such as those encoding for tail and capsid proteins, homologous to any previously characterized phage structural proteins, were not found and further protein expression studies would be required to fully elucidate the functional roles of many of the ϕBrb01 and ϕBrb02 genes.

Of the five phage genomes described in this study, the genome sequence of the lytic phage ϕSb01 was found to have the greatest homology to previously sequenced prophages and lytic phages infecting bacteria of the *Streptococcus* genus. This greatly facilitated annotation of the phage genome and the functional, modular organization of the genome could be determined. Genes for phage integration and maintenance of lysogeny were absent from the genome indicating that this phage relied on the lytic cycle of phage replication to persist in the rumen environment. This lytic phage could also infect and successfully replicate in multiple hosts, being originally isolated with the ovine rumen isolate *S. equinus* 2B as host strain and later shown to also infect two additional bovine rumen isolates of *S. equinus* (strains Sb04 and Sb17) (Klieve and Bauchop, 1991; Klieve et al., 1999). This indicates that this phage uses cell wall receptors that are relatively common in *S. equinus* for initial phage attachment and encodes for proteins which can function in closely-related bacterial strains, including those of both ovine and bovine origin. Phylogenetic analysis on the basis of the *TerL* gene, and comparative analysis of predicted phage proteins, indicated that this phage was highly related to phages and prophages known to infect other *Streptococcus* species, including those of *S. pyogenes, S. parauberis*, and *S. agalactiae*. Modules of genes contained in related phages were also present in the ϕSb01 genome, suggesting an evolutionary history of genetic exchange and conservation of phage gene modules, which may facilitate the ability to replicate in a wider spectrum of *Streptococcus* hosts and contribute to an increased phage host range.

The availability of bacterial genome assemblies for the phage host strains has contributed to our understanding of phage:host interactions, enabling an assessment of the phage defense systems employed by these bacteria. Previous studies have noted the formation of extracellular polysaccharide capsules and clumping of cells contributing to host resistance to the lytic phages ϕBrb01 and ϕSb01 (Klieve and Bauchop, 1991; Klieve et al., 1991). Interestingly, whatever physical and enzymatic mechanisms the respective bacterial hosts employ to prevent phage attachment and intracellular replication, the lytic phages must be either unaffected by or be able to avoid or circumvent these mechanisms, in order to successfully replicate.

While the phages ϕBrb01 and ϕBrb02 were previously shown to infect three and four rumen-derived *Bacteroides* isolates (Klieve et al., 1991) respectively, only one of these bacterial strains has been genome sequenced (*Bacteroides* sp. AR20). Genomes of the rumen *Bacteroides* and *Ruminococcus* host strains AR20 and AR67 did not contain intact prophage elements and although short CRISPR regions were detected, these strains appeared to lack *Cas* proteins. The genomes of these bacteria however, did contain an extensive diversity and numbers of genes relating to RM systems, suggesting that these bacteria rely more on these mechanisms for specific and non-specific methylation and/or cleavage of incoming, foreign DNA and consequently protect against phage infection. In contrast the three *Streptococcus* genomes (strains 2B, Sb04, and Sb17) contained fewer genes relating to Type II, Type IIG, and Type IV RM systems than the *Bacteroides* and *Ruminococcus* genomes. The complex Type I RM systems identified in the *strains* Sb04 and Sb17 were absent from the genome of strain 2B. In addition, the genome for strain 2B was the only bacterial host found to contain a possibly intact prophage element (designated ϕSb2Bpro1). This prophage element did not appear to convey super-infection immunity to the lytic phage ϕSb01 and the lack of CRISPR elements and relatively low numbers of genes relating to RM systems, may explain the previous observation that this *Streptococcus* strain may be more receptive to lytic and lysogenic phage infection than other rumen-derived *Streptococcus* isolates (Klieve et al., 1999). The variety and extent of the phage defense strategies utilized by the five different bacteria examined in this study are likely to reflect those employed in the wider rumen microbial community, indicating that some bacterial strains, even within the same genus, may be more receptive to phage infection and sustaining phage replication than others.

With improved methods for DNA sequencing and the advent of metagenomic studies to comprehensively sequence phage particle fractions obtained from rumen fluid, the full extent of viral diversity within the rumen is gradually being revealed. Exploring metagenomic datasets relies on sequence homology to classify the large volumes of sequence information generated and explorations of the rumen viral metagenome have indicated extensive sequence homology to Caudovirales-like lytic phages and prophage elements originating from rumen bacteria and bacteria from other environments (Berg Miller et al., 2012; Ross et al., 2013). The genome sequencing of phage isolates that infect common gastrointestinal bacteria will complement and greatly enhance the accuracy of phage classification and bioinformatics analysis of viral metagenomics datasets, providing reference sequences and novel viral genes to which relevant sequence homology can be conferred.

## Author contributions

RG, SL, WK, EA, DO, and AK: Contributed to the conception and design of the work; RG, CM, and DO: Contributed to the preparation of DNA; WK and SL: Contributed to aquisition of data; RG, WK, and EA: Contributed to data analysis. All authors contributed to the interpretation of data, critical revision and final approval of the version to be published. All authors are in agreement to be accountable for all aspects of the work in ensuring that questions related to the accuracy or integrity of any part of the work are appropriately investigated and resolved.

### Conflict of interest statement

The authors declare that the research was conducted in the absence of any commercial or financial relationships that could be construed as a potential conflict of interest.
